# A Fluorescence-Based High-Throughput Assay for the Discovery of Exchange Protein Directly Activated by Cyclic AMP (EPAC) Antagonists

**DOI:** 10.1371/journal.pone.0030441

**Published:** 2012-01-19

**Authors:** Tamara Tsalkova, Fang C. Mei, Xiaodong Cheng

**Affiliations:** Department of Pharmacology and Toxicology and Sealy Center for Structural Biology and Molecular Biophysics, The University of Texas Medical Branch, Galveston, Texas, United States of America; Universidade Federal do Rio de Janeiro, Brazil

## Abstract

**Background:**

The discovery, more than ten years ago, of exchange proteins directly activated by cAMP (EPAC) as a new family of intracellular cAMP receptors revolutionized the cAMP signaling research field. Extensive studies have revealed that the cAMP signaling network is much more complex and dynamic as many cAMP-related cellular processes, previously thought to be controlled by protein kinase A, are found to be also mediated by EPAC proteins. Although there have been many important discoveries in the roles of EPACs greater understanding of their physiological function in cAMP-mediated signaling is impeded by the absence of EPAC-specific antagonist.

**Methodology/Principal Findings:**

To overcome this deficit, we have developed a fluorescence-based high throughput assay for screening EPAC specific antagonists. Our assay is highly reproducible and simple to perform using the “mix and measure” format. A pilot screening using the NCI-DTP diversity set library led to the identification of small chemical compounds capable of specifically inhibiting cAMP-induced EPAC activation while not affecting PKA activity.

**Conclusions/Significance:**

Our study establishes a robust high throughput screening assay that can be effectively applied for the discovery of EPAC-specific antagonists, which may provide valuable pharmacological tools for elucidating the biological functions of EPAC and for promoting an understanding of disease mechanisms related to EPAC/cAMP signaling.

## Introduction

cAMP-mediated signaling regulates a myriad of important biological processes under both physiological and pathological conditions. In multi-cellular eukaryotic organisms, the effects of cAMP are transduced by two ubiquitously-expressed intracellular cAMP receptors, the classic protein kinase A/cAMP-dependent protein kinase (PKA/cAPK) and the more recently discovered exchange protein directly activated by cAMP/cAMP-regulated guanine nucleotide exchange factor (EPAC/cAMP-GEF) [1], [2]. Since both PKA and EPAC are ubiquitously expressed in all tissues, an increase in intracellular cAMP levels will lead to the activation of both PKA and EPAC. Net physiological effects of cAMP entail the integration of EPAC- and PKA-dependent pathways in a spatial and temporal manner. Depending upon their relative abundance, distribution and localization, as well as the precise cellular environment, the two intracellular cAMP receptors may act independently, converge synergistically, or oppose each other in regulating a specific cellular function [3]. Therefore, careful dissections of the individual role and relative contribution of EPAC and PKA within the overall cAMP signaling in various model systems are critical for further elucidating the mechanism of cAMP signaling, as well as essential for developing novel mechanism-based therapeutic strategies targeting specific cAMP-signaling components.

Selective pharmacological probes, particularly inhibitors, have been valuable tools for dissecting the physiological functions of signaling molecules and the mechanism of signal transduction pathways. Over the years, the cAMP analog, 8-(4-chloro-phenylthio)-2′-O-methyladenosine-3′,5′-cyclic monophosphate (8-CPT-2′-O-Me-cAMP/007), and its derivatives that selectively activate EPAC over PKA have been developed based on structure/sequence alignment analysis [4], [5]. 8-CPT-2′-O-Me-cAMP exerts about 100-fold selectivity towards EPAC over PKA and has become a widely used tool in EPAC-related research [4]–[9]. Limitations of the 8-CPT-2′-O-Me-cAMP class of compounds include low membrane permeability and poor cellular potency [10], [11]. Recently, a caged 8-CPT-2′-O-Me-cAMP derivative, 8-CPT-2′-O-Me-cAMP-AM, with enhanced membrane permeability has been developed [10], [11]. Despite this significant improvement, the biological applications of 8-CPT-2′-O-Me-cAMP -related compounds are limited by their off-target effects inhibiting phosphodiesterases (PDEs) in the cell, which causes elevation of cAMP or/and cGMP and therefore indirect activation of PKA, PKG and/or cyclic nucleotide gated channels [12]. So far, no EPAC-specific antagonists have been reported, and developing EPAC-specific pharmacological probes to dissect the physiological functions that EPAC play in the overall cAMP-mediated signaling remains a major challenge within the research field. To bridge this major gap in our knowledge, we have developed a robust high throughput assay for the purpose of identifying small pharmacological probes that are capable of inhibiting EPAC functions *in vitro*.

## Results

### Assay readout and signal intensity

**Figure 1:**
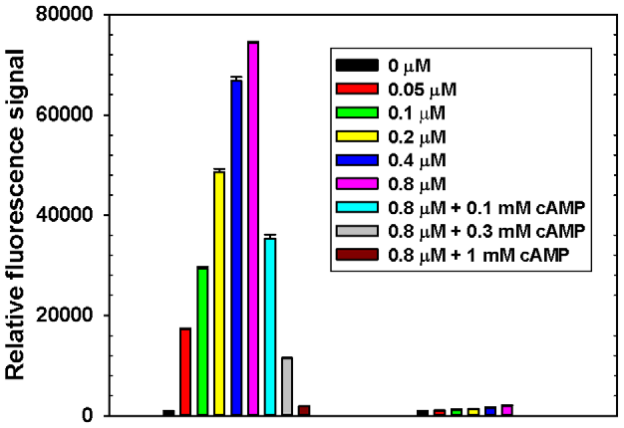
Binding of 8-NBD-cAMP to EPAC1 and 2. (A) Change in fluorescence when 8-NBD-cAMP (0.1 µM) is titrated with EPAC1 or EPAC2. The fluorescence change can be reversed by the addition of excess cAMP. Data are from three independent experiments with error bars representing standard deviations.

To develop a sensitive and robust HTS assay for EPAC, we decided to use fluorescent cyclic nucleotide analogs, which have been used extensively to probe the interactions between cyclic nucleotide and its receptors. Among all the fluorescent cAMP analogs that we tested, 8-NBD-cAMP (8-(2-[7-Nitro-4-benzofurazanyl] aminoethyl-thio) adenosine-3′, 5′-cyclic monophosphate), gave the largest fluorescent change when titrated with purified full-length EPAC2. As shown in **Figure 1**, the intrinsic fluorescence of 8-NBD-cAMP, alone in solution, is very low. Binding of 8-NBD-cAMP to EPAC2 led to a dose-dependent increase in fluorescent signal. A more than 100 fold increase in 8-NBD-cAMP fluorescent signal was observed under near saturating EPAC2 concentration. The fluorescence change seen with 8-NBD-cAMP can be reversed by the addition of excess unlabelled cAMP, which competes with 8-NBD-cAMP in binding to EPAC2. On the other hand, the magnitude of fluorescence increase associated with EPAC1 binding was much more modest, which is consistent with the finding that binding of 8-NBD-cAMP to an isolated fragment of the cAMP binding domain of EPAC1 leads to a maximal six-fold increase in fluorescence intensity [13]. This very large reversible fluorescence change of 8-NBD-cAMP during its binding to EPAC2 protein made it an excellent readout for designing a sensitive and robust HTS assay for EPAC antagonists. To define the suitable protein and ligand concentrations for our screen, we tested the concentration-dependence of the fluorescence signals in 96-well format by fixing the ratio of EPAC2/8-NBD-cAMP at 1∶1.2. As shown in **Figure S1**, the fluorescence intensity signal showed an excellent linearity as a function of EPAC2 concentration. We observed a signal-to-background ratio of 7 even at 0.025 µM of EPAC2 concentration. On the basis of these results, concentrations at 0.05 µM and 0.06 µM for EPAC2/8-NBD-cAMP, respectively, were used for subsequent experiments in 96-well format.

### Overall quality of the assay in 96-well format

To determine the suitability or quality of our proposed assay for HTS, we performed preliminary studies in 96-well plates. 100 µl of EPAC2/8-NBD-cAMP (0.05 µM/0.06 µM) was manually pipetted into each well of the plate and fluorescent signals were then monitored using a Molecular Devices SpectraMax M2 microplate reader. The background (control) signal of each well was measured in the excess of cAMP (300 µM). Our assay showed an excellent separation between sample and control signals (**Figure S2)**. To determine the reproducibility, between-plate and day-to-day variations of the assay, independent tests were performed. As demonstrated in **Table 1**, all major statistical parameters showed excellent match between independent experiments. The calculated Z′ scores were above 0.8. These results suggest that our assay is highly reproducible with little between-plate and day-to-day variations. To test the effect of DMSO, we measured the fluorescent signal of EPAC2/8-NBD-cAMP in the presence of various concentration of DMSO. As shown in **Table 2**, the measured florescence signal in the presence of 1% DMSO was within 5% of that in the absence of DMSO. Therefore, our assay is well tolerated up to 1% of DMSO.

**Table 1:** Summary of assay statistic parameters* of independent experiments.

Experiments	S	σ_S_	CV (%)	B	σ_B_	S/B	S/N	Z′
Exp. 1	11,686	516	4.4	1,488	103	7.8	101.9	0.82
Exp. 2	11,016	375	3.3	1,526	81	7.2	105.4	0.85

*S: mean signal; σ_S_: standard deviation of signal; CV: coefficient of variation; B: mean background (control); σ_B_: standard deviation of background; S/B: signal-to-background ratio; S/N: signal-to-noise ratio; Z′: Z′ score as determined by 
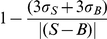
 [21].

**Table 2:** Fluorescent signals of EPAC2/8-NBD-cAMP as a function of DMSO concentration.

[DMSO] (%)	0	0.1	0.2	0.5	1.0
**Signal (%)**	100.0±1.8	100.9±2.1	99.9±3.5	96.2±0.9	96.2±3.6

### Dose-dependent responses to cAMP analogs

**Figure 2:**
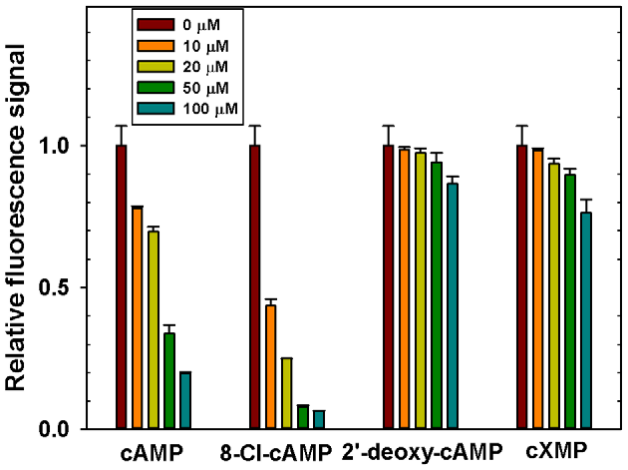
Dose-dependent response of selected cAMP analogs of known activity. Active compounds: cAMP and 8-Cl-cAMP; inactive compounds: 2′-deoxy-cAMP and cXMP. Data are from three independent experiments with error bars representing standard deviations.

To further evaluate our assay, we determined the dose responses of a small collection of known cAMP analogs: cAMP and 8-Cl-cAMP can bind and activate EPAC while 2′-deoxy-cAMP and cXMP bind EPAC weakly and are incapable of activating EPAC. As expected, cAMP and 8-Cl-cAMP led to significant dose-dependent inhibitions of fluorescence signals while 2′-deoxy-cAMP and cXMP had minimal effects (**Figure 2**). Moreover, the degree of signal decrease is consistent with the relative affinity of these analogs to EPAC. For example, 8-Cl-cAMP led to a bigger decrease in signal than cAMP at the same compounds concentrations as 8-Cl-cAMP binds EPAC tighter than cAMP. Based on these results, we chose a single-dose compound concentration of 100 µM, at which concentration cAMP resulted in a ∼80% decrease in fluorescence signal, for HTS screening. Compounds that cause more than 80% decrease in NBD fluorescence intensity at 100 µM concentration are selected as initial positive hits and evaluated further using secondary and counter-screening assays.

#### Screening of EPAC antagonists using a NCI-diversity set library

**Figure 3:**
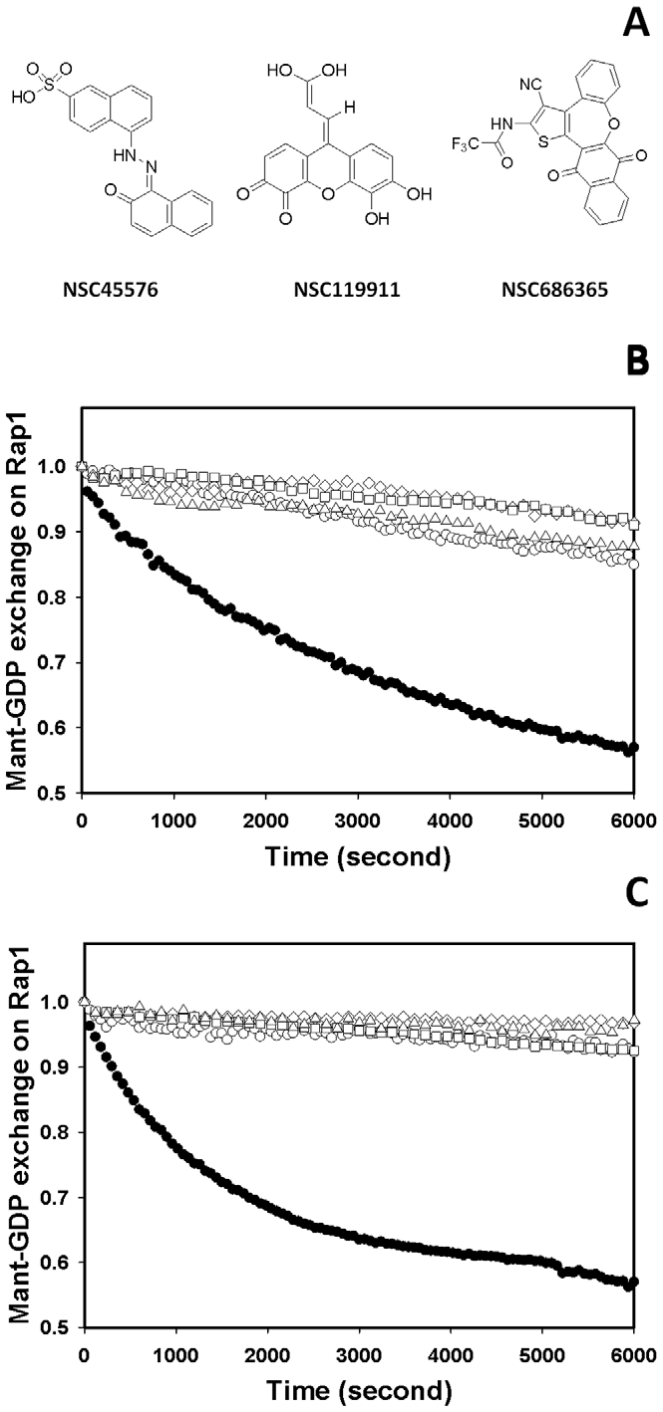
Identification of EPAC antagonists from HTS screen. (A) Chemical structures of identified EPAC inhibitors. cAMP-mediated EPAC2 (B) or EPAC1 (C) GEF activity measured in the presence or absence of EPAC antagonists: open circles, EPAC alone; closed circles: EPAC in the presence of 25 µM cAMP; open squares, EPAC with 25 µM cAMP and 25 µM NSC45576; open diamonds, EPAC with 25 µM cAMP and 25 µM NSC119911; and open triangles up, EPAC with 25 µM cAMP and 25 µM NSC686365. Similar results were obtained from three independent experiments.

As a validation step for translation of the assay to HTS, we performed a pilot screening in 96-well format using the NCI DTP (Developmental Therapeutics Program) diversity set library. The NCI diversity set contains 1990 carefully selected small molecules for their high chemical and pharmacological diversity from the entire collection of 140,000 compounds at NCI. Majority of the compounds did not affect the binding of 8-NBD-cAMP to EPAC2 and about 18 compounds decreased the fluorescence signal more than 80% at a test concentration of 100 µM. These 18 compounds were further tested individually using a secondary functional assay that monitors the ability of EPAC to catalyze the nucleotide exchange activity of Rap1. Three compounds NCS45576, NCS119911 and NSC686365 were shown to be able to inhibit EPAC2 GEF activity to basal levels at 25 µM concentration in the presence of equal concentration of cAMP (**Figure 3A&B**). Since these hits were obtained using an assay developed against EPAC2, the ability of these compounds to inhibit EPAC1 GEF activity was also tested in parallel. All three compounds were able to inhibit EPAC1-mediated Rap1 nucleotide exchange at 25 µM concentration in the presence of equal concentration of cAMP (**Figure 3C**).

**Figure 4:**
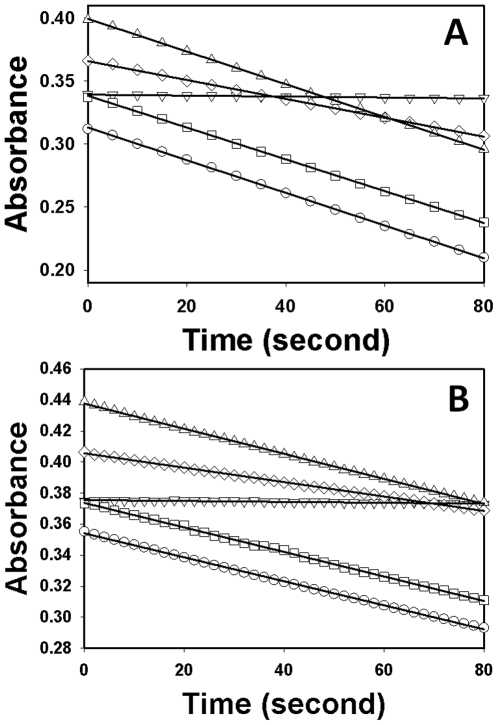
Effects of NSC45576 and NSC119911 on PKA activities. Type I (A) and II (B) PKA holoenzyme activities in the presence of 0.1 mM cAMP: open circles, control; open triangles down, 25 µM H89; open squares, 25 µM NSC45576; open diamonds, 25 µM NSC119911; and open triangles up, 25 µM NSC686365. Similar results were obtained from three independent experiments.

To test the specificity of these compounds, we performed counter-screening assays that measure type I and II PKA holoenzyme activities, respectively. As shown in **Figure 4**, 25 µM of NCS45576 and NSC686365 did not significantly alter cAMP-induced type I and II PKA holoenzymes activation while NCS119911 blocked ∼50% of type I or II PKA activities, respectively. These results suggest that NCS45576 and NSC686365 are EPAC specific inhibitors (ESI) that selectively block cAMP-induced EPAC activation but do not inhibit cAMP-mediated PKA activation.

**Figure 5:**
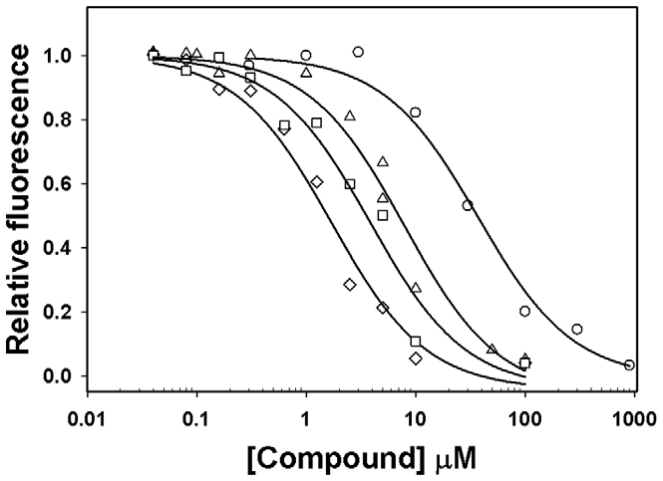
Relative potency of identified EPAC antagonists. Dose-dependent competition of EPAC antagonists with 8-NBD-cAMP in binding to EPAC2: open circles, cAMP control; open squares, 25 µM NSC45576; open diamonds, 25 µM NSC119911; and open triangles up, 25 µM NSC686365. Similar results were obtained from three independent experiments.

To further characterize the relative potency of these indentified EPAC antagonists, we determined the apparent IC_50_ of these compounds in competing of 8-NBD-cAMP binding. As revealed by **Figure 5**, NSC45576, NSC119911 and NSC686365 competed with 8-NBD-cAMP binding with apparent IC_50_ of 1.7, 3.8 and 7.9 µM, respectively, while cAMP competed with 8-NBD-cAMP binding with a IC_50_ of 40 µM. Taken together, we have established a robust HTS assay that is capable of identifying small chemical probes specific for EPAC and not cross-reacting with PKA.

## Discussion

In this study, we developed a sensitive high throughput assay for screening EPAC specific pharmacological probes utilizing the environmentally-sensitive nitrobenzoxadiazole (NBD) fluorophore. The assay takes advantage of the fact that the weakly fluorescent 8-NBD-cAMP becomes brilliant fluorescently when it binds to EPAC2. This large increase in fluorescent intensity allows us to adapt the assay for high throughput screening using very low EPAC2 and 8-NBD-cAMP concentrations. Furthermore, the HTS assay can be performed effectively and simply using the “mix-and-measure” format with high reproducibility. When performed pilot screen under the 96-well format, our screening assay routinely provided an excellent Z′ score above 0.8.

As a validation step for translation of the assay to HTS, we performed a pilot screen using the NCI DTP (Developmental Therapeutics Program) diversity set library. The NCI diversity set contains 1990 carefully selected small molecules for their high chemical and pharmacological diversity from the entire collection of 140,000 compounds at NCI. Our primary screening resulted in an initial hit rate less than 1%. Subsequent validation using individually ordered compounds and a secondary confirmation assay that measured the guanine nucleotide exchange activity of EPAC led to the identification of two EPAC antagonists. The design of assay is particularly suitable for searching compounds that directly compete with 8-NBD-cAMP in binding to EPAC2. However, in theory if a chemical library with a large enough size is used, the assay should also be capable of identifying allosteric effectors that lock EPAC2 in conformations unfavorable for 8-NBD-cAMP binding but are not in direct competition with probe. Therefore, with its high sensitivity and broad dynamic range, our assay should be able to be easily adapted to higher throughput formats so allosteric inhibitors or even activators of EPAC can be identified.

It should be emphasized that the assay described in this study represents an initial effort in identifying EPAC-specific antagonists. Before small molecules identified using this assay can be utilized as pharmacological probes for dissecting the physiological functions of EPAC proteins, their pharmacological properties should be carefully evaluated further to ensure that they don't disrupt other components of the cyclic nucleotide signaling circuits. For example, while we have tested the effect of our compounds on PKA, an immediately related cAMP signaling molecule, their effects on other peripheral signaling molecules in cells related to the EPAC pathway such as phosphodiesterases or adenylyl cyclases should also be examined.

## Materials and Methods

### Reagents

8-NBD-cAMP, cXMP, 8-Cl-cAMP and 2′-deoxy-cAMP were purchased from BioLog Life Science Institute (Bremen, Germany). MANT-GDP, a fluorescent GDP analog, was obtained from Invitrogen (Carlsbad, CA, USA). All other reagents were purchased through Sigma-Aldrich (St. Louse, MO, USA).

### Compound libraries

Small chemical compounds from the Diversity set (1990 compounds), were obtained from the Open Chemical Repository of National Cancer Institute Developmental Therapeutics Program (DTP). Compounds were supplied in DMSO in 96-well polypropylene plates and stored at −80°C. Replica daughter plates were generated by diluting original stock plates 10-fold in DMSO and used for screening.

### Protein expression and purification

Recombinant EPAC1, EPAC2 and C-terminal truncated Rap1B(1-167) were purified as described previously [14]–[16]. PKA RIα, RIIβ and catalytic subunits were recombinantly expressed in *E. coli* and purified to homogeneity as reported [17]. Type I and II PKA holoenzymes were reconstituted from individually purified recombinant PKA R and C subunits [18] All proteins used in this study were at least 95% pure, as judged by SDS PAGE.

### Primary screen assay

Fluorescence intensity of 8-NBD-cAMP in complex with EPAC2 has been used as the readout in the primary screen assay. Primary screen of NCI DTP (Developmental Therapeutics Program) diversity set library was performed in black 96-well microplates from Corning Costar (Cambridge, MA, USA). Briefly, 50 nM EPAC2 solution was prepared in 20 mM Tris buffer, pH 7.5, containing 150 mM NaCl, 1 mM EDTA and 1 mM DDT. 8-NBD-cAMP was added to EPAC2 solution up to 60 nM from 17 µM stock solution in water. Sample has been dispensed into 96-well plate (100 µl/well) and test compounds were added (1 µl/well) from 96-well mother plates. Test compounds were added from 10 mM stock solutions in DMSO. Samples with cAMP addition (1 µl/well from 30 mM stock solution in water) and no additions have been used as a positive and a negative control. Fluorescence intensity signal from 8-NBD probe was recorded at room temperature before and after tested compounds were added using SpectaMaxM2 microplate reader (Molecular Devices, Silicon Valley, CA, USA) with excitation/emission wavelengths set at 470/540 nm.

### Secondary confirmation assay

Measurement of in vitro guanine nucleotide exchange factor (GEF) activity of EPAC was adapted from a well known fluorescence-based assay using a fluorescent guanine nucleotide analog [19], and used as a functional confirmation assay for the compounds identified from primary screen. Briefly, 0.2 µM of Rap1B(1–167) loaded with the fluorescent GDP analog (Mant-GDP), was incubated with EPAC in 50 mM Tris buffer pH 7.5, containing 50 mM NaCl, 5 mM MgCl_2_, 1 mM DTT and a 100-fold molar excess of unlabeled GDP (20 µM) in the presence of 25 µM tested compound and 25 µM cAMP. Exchange of Mant-GDP by GDP was measured as a decrease in fluorescence intensity over time using a FluoroMax-3 spectrofluorometer with excitation/emission wavelengths set at 366/450 nm. Typically, decay in the fluorescence intensity was recorded over a time course of 6000 s with data points taken every 60 s.

### Counter screening assay

Kinase activities of the type I and II PKA holoenzymes were measured spectrophotometrically in a 96-well plate with a coupled enzyme assay as described previously [20]. In this assay, the formation of the ADP is coupled to the oxidation of NADH by the pyruvate kinase/lactate dehydrogenase reactions so the reaction rate can be determined by following the oxidation of NADH, reflected by a decrease in absorbance at 340 nm. The kinase reaction mixture (100 µl) contained 50 mM Mops (pH 7.0), 10 mM MgCl_2_, 1 mM ATP, 1 mM PEP, 0.1 mM NADH, 8 U of pyruvate kinase, 15 U of lactate dehydrogenase, fixed amount of type I or type II PKA holoenzyme and 0.1 mM cAMP, with or without 25 µM of test compound. Reactions were pre-equilibrated at room temperature and initiated by adding the Kemptide substrate (final concentration 0.26 mM). PKA activities measured in the presence of 25 µM H89, a selective PKA inhibitor, were used as positive controls of PKA inhibition.

## Supporting Information

Figure S1 **Fluorescence intensities of Epac2/8-NBD-cAMP as a function of protein concentrations.** Fluorescence signals of Epac2/8-NBD-cAMP (filled circles) and 8-NBD-cAMP (open circles) alone measured in a 96-well plate.
(TIF)

Figure S2 **Typical assay data from a test run in 96-well format.** The solid horizontal lines show the means of the sample of 0.05/0.06 µM of Epac2/8-NBD-cAMP (filled circles) and background data in the presence of 300 µM of cAMP (open circles). Broken lines display 3 stardard deviations (SD) from the mean of each date set.
(TIF)
