# Supplementary figures and images for: A Fluorescence-Based High-Throughput Assay for the Discovery of Exchange Protein Directly Activated by Cyclic AMP (EPAC) Antagonists
Source: PLoS One. 2012 Jan 19;7(1):e30441. doi: 10.1371/journal.pone.0030441 (PMC3262007; doi:10.1371/journal.pone.0030441)

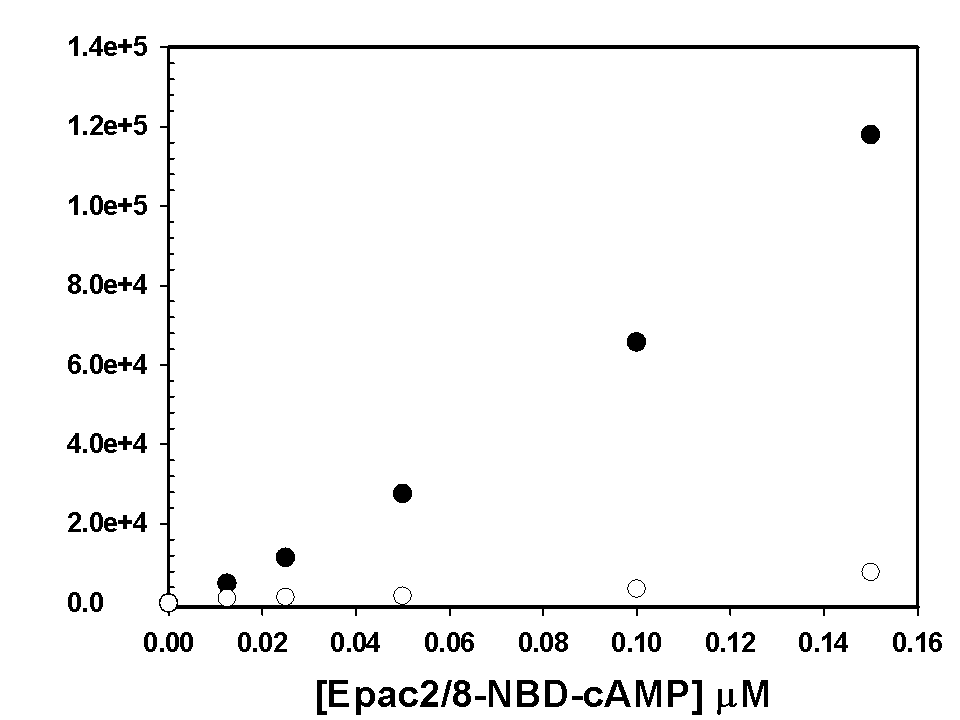

Supplement: Figure S1 — Fluorescence intensities of Epac2/8-NBD-cAMP as a function of protein concentrations. Fluorescence signals of Epac2/8-NBD-cAMP (filled circles) and 8-NBD-cAMP (open circles) alone measured in a 96-well plate. (TIF) [file pone.0030441.s001.tif]

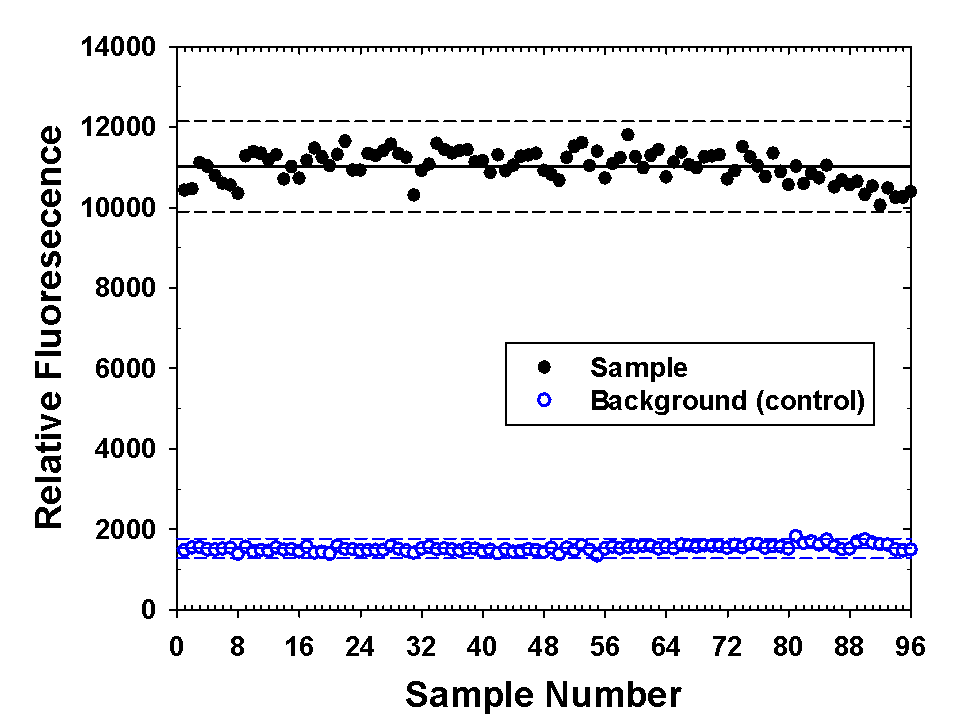

Supplement: Figure S2 — Typical assay data from a test run in 96-well format. The solid horizontal lines show the means of the sample of 0.05/0.06 µM of Epac2/8-NBD-cAMP (filled circles) and background data in the presence of 300 µM of cAMP (open circles). Broken lines display 3 stardard deviations (SD) from the mean of each date set. (TIF) [file pone.0030441.s002.tif]
